# Variation in Tacrolimus Trough Concentrations in Liver Transplant Patients Undergoing Endoscopic Retrograde Cholangiopancreatography: A Retrospective, Observational Study

**DOI:** 10.3389/fphar.2020.01252

**Published:** 2020-08-19

**Authors:** Rongrong Wang, Weili Wang, Kuifen Ma, Xin Duan, Fangfang Wang, Mingzhu Huang, Wei Zhang, Tingbo Liang

**Affiliations:** ^1^ Department of Clinical Pharmacy, the First Affiliated Hospital, College of Medicine, Zhejiang University, Hangzhou, China; ^2^ Department of Hepatobiliary and Pancreatic Surgery, The First Affiliated Hospital, Zhejiang University School of Medicine, Hangzhou, China; ^3^ Department of Pharmacy, Children’s Hospital, Zhejiang University School of Medicine, Hangzhou, China

**Keywords:** liver transplant, tacrolimus, trough concentration, endoscopic retrograde cholangiopancreatography, related factors

## Abstract

**Objective:**

High variabilities in tacrolimus (TAC) exposure are still problems that confuse physicians. TAC trough levels (TAC C_min_) fluctuated considerably after endoscopic retrograde cholangiopancreatography (ERCP) treatment in several liver transplant (LT) patients. We aimed to investigate the variation regularity of TAC C_min_ post-ERCP and related factors.

**Methods:**

This study was a retrospective, observational study conducted at the First Affiliated Hospital of Zhejiang University in China. From October 2017 to January 2019, 26 LT patients that received ERCP were included (73 TAC C_min_ measures). The absolute difference and the variation extent in TAC C_min_ pre- and post-ERCP were analyzed. Patients were divided into mild and obvious variation groups, and the differences were compared.

**Results:**

The TAC C_min_ in LT patients significantly increased in the first three days post-ERCP (*p*<0.05) and increased by more than 20% in 18 out of 26 (69.2%) patients. The mean extent of variation in TAC C_min_ was 45.1% (95% confidence interval [CI]: 28.3–81.3%) and 31.4% (95% CI: 9.7–53.1%) on days 1 and 3 post-ERCP, respectively. The increasing TAC C_min_ gradually returned to baseline within a week (*p*>0.05). The daily TAC dose and total bile acid (TBA) level were significantly higher (*p*<0.05) in patients with obvious variation in TAC C_min_. The differences in other demographics, clinical characteristics, variation in laboratory data, and serum amylase levels between the two groups were not significant.

**Conclusion:**

The TAC C_min_ significantly increased in LT patients during the first three days after ERCP, and the level returned to baseline within a week. The daily TAC dose and TBA levels may be related to this increase. Frequent drug concentration monitoring should be executed in the early phase post-ERCP, especially in patients with related factors.

## Introduction 

Tacrolimus (TAC) was approved by the USA Food and Drug Administration as an immunosuppressive regimen for liver transplantation (LT) in 1994. TAC improves the outcomes of LT significantly; thus, it has been used as a first-line treatment for LT recipients (EASL, 2016; Charlton et al., 2018; Brunet et al., 2019). TAC has a narrow therapeutic index and high interindividual and intraindividual variabilities in pharmacokinetics (Christina et al., 2014; Defrancq et al., 2019). The insufficient exposure of TAC increases the risk of rejection, whereas overexposure increases the occurrence of adverse effects, such as hyperkalemia, hypertension, nephrotoxicity, and dyslipidemia (Sheiner et al., 2000; Kuo et al., 2010; Watt and Charlton, 2010; Charlton et al., 2018). High variability in TAC is associated with TAC-related toxicity and poor survival in LT patients and other solid organ transplantations (Shuker et al., 2015; Shemesh et al., 2017; Del Bello et al., 2018; Rayar et al., 2018; van der Veer et al., 2019; Kuypers, 2020). Therefore, therapeutic drug monitoring (TDM) of TAC is routinely conducted to maintain the trough concentration of TAC (TAC C_min_) within the therapeutic range (Charlton et al., 2018; Brunet et al., 2019). Several dosing algorithms were developed to predict the dose requirement of TAC on the basis of defined clinical factors and demographic characteristics (Vanhove et al., 2016). Despite the appropriate use of TDM and these algorithms, high inter-patient and intra-patient variabilities in TAC exposure caused by complex or unknown factors are still problems that confuse physicians (Shuker et al., 2015; Shemesh et al., 2017; Del Bello et al., 2018; Kuypers, 2020). Therefore, unknown factors that can considerably interfere with the pharmacokinetic process of TAC should be determined.

Factors such as mealtime, food, and drug interactions have been studied (Vanhove et al., 2016). However, few studies have examined the variation in TAC exposure during surgery or interventional procedures. Biliary complications are the most frequent complications following orthotopic LTs (Dai et al., 2017; Martins et al., 2018; Sendino et al., 2018), and their incidence rates are between 5–32% (Tringali et al., 2016). Endoscopic retrograde cholangiopancreatography (ERCP) therapy is the first-line treatment strategy for the management of biliary complications. We occasionally found that the TAC C_min_ increased obviously in some LT patients post-ERCP, but no study has reported this phenomenon. Therefore, we conducted a retrospective, observational study to investigate the variation regularity of TAC C_min_ post-ERCP and potential risk factors underlying this process.

## Method

### Study Design and Participants

This work was a retrospective, observational study conducted in the LT center of the First Affiliated Hospital of Zhejiang University (FAHZJ) in China. FAHZJ is a university-affiliated tertiary hospital with 2,500 beds and has one of the largest LT centers nationwide. LT recipients who underwent ERCP between October 2017 and January 2019 were enrolled. The inclusion criteria were as follows: 1) LT recipients who received fixed doses of TAC pre-ERCP; 2) patients who did not take potentially interacting concomitant medications; 3) patients whose serum TAC C_min_ values were tested within three days pre-ERCP and post-ERCP; 4) patients with no diarrhea pre-ERCP; 5) If a patient underwent ERCP several times during the study period, then only the first set of eligible data were included. If a patient had diarrhea post-ERCP, the related serum TAC C_min_ would be excluded.

In our center, patients were asked to fast for about 24 h post-ERCP, and to take TAC at scheduled times during hospitalization. Immediate-released tacrolimus was used twice daily at fixed times. TAC should be targeted to a C_min_ of 3–7 ng/ml during different phases after LT. Our nursing staff and pharmacists will guide therapeutic strategies for patients daily during hospitalization to keep their adherence. The study was conducted in accordance with the Helsinki Declaration of 1975 and followed the statement of Strengthening the Reporting of Observational Studies in Epidemiology. Ethical approval was obtained from the authorized ethics committee of FAHZJ (IIT20200321A).

### Data Collection

We reviewed the clinical electronic medical records and collected the demographic, clinical, laboratory, and outcome data of all the included patients. A standard case report form was used to record data, including sex, age, weight, laboratory data, LT time, ERCP types, indication for ERCP, immunosuppressive regimes, daily TAC dose, and TAC C_min_. Missing data were obtained through direct communication with the patients and their families, as well as with doctors responsible for the patients’ treatment. All data were clarified by three researchers.

### Outcomes

The primary outcomes were the absolute difference in TAC C_min_ pre- and post-ERCP and the extent of variation in TAC C_min_.

Absolute difference in TAC C_min_ _(n days post-ERCP)_ = TAC C_min (n days post-ERCP)_ –TAC C_min_ _(pre-ERCP)_. The extent of variation in TAC C_min (n days post-ERCP)_ (%) = (TAC C_min_ _(n days post-ERCP)_ – TAC C_min_ _(pre-ERCP)_)/TAC C_min (pre-ERCP)_ ×100. According to the extent of variation in TAC C_min_ within three days post-ERCP, patients were categorized into two groups, namely, mild variation group (extent of variation in TAC C_min_ <20%), and obvious variation group (extent of variation in TAC C_min_ ≥20%).

The secondary outcomes included the differences in demographics and clinical characteristics and variation in laboratory data (liver function indicators, electrolyte levels, uric acid, serum creatinine, blood glucose, and serum amylase levels) between patients with or without obvious variation in TAC C_min_.

### Statistical Analysis

Categorical variables were expressed as numbers (percentages), whereas continuous variables were expressed as means (standard deviations [SD]) or medians (interquartile range [IQR]), appropriately. The variation in TAC C_min_ was analyzed utilizing paired-samples *t*-test. Demographic data, clinical characteristics, and variation of laboratory data were compared between patients with or without obvious variation in TAC C_min_ through Student’s *t*-test. Pearson χ2 or Fisher exact test (cell size<5) was used to compare the frequency distribution of the categorized parameters. All analyses were performed with the application of SPSS software (SPSS Inc., Chicago, IL). Statistical significance was defined as two-sided *p*<0.05.

### TAC Trough Level Assays

The whole blood trough levels of TAC were measured by the enzyme multiplied technique (Emit^®^2000 TAC Assay, Unite State, 8R019UL), which is routinely used to determine TAC levels in clinical practice (Gounden and Soldin, 2014; Kalt, 2017).

## Result

### Baseline of Included Patients

**Figure 1:**
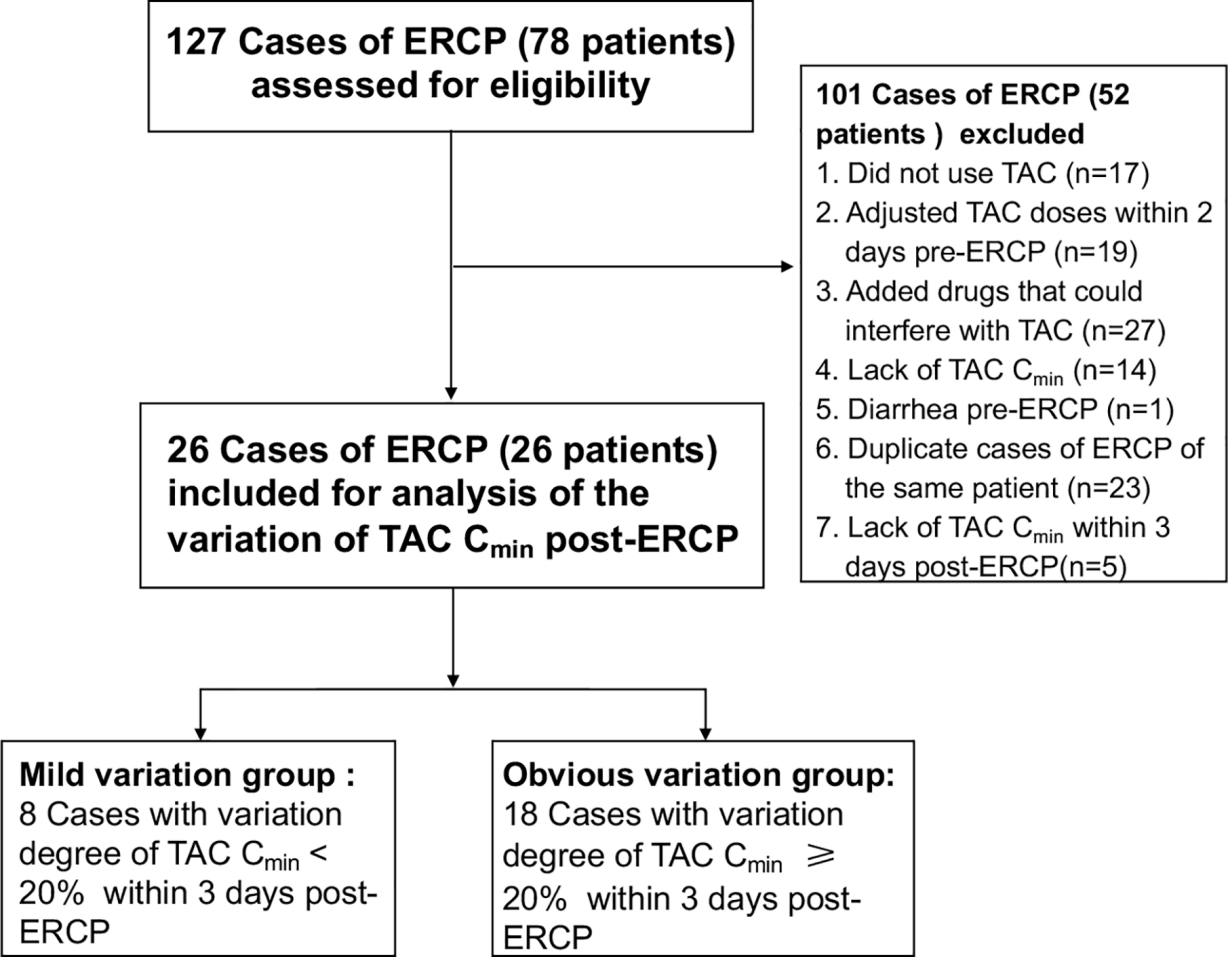
Study flow diagram.

From October 2017 to January 2019, 26 LT patients (**Figure 1**) were enrolled in the study, among which 88.5% were male. The mean age was 48.7 years (SD: 11.1), and the median duration since LT was 10.0 months (IQR: 2.8–44.3). The indications for ERCP were anastomotic stricture in 88.5% of patients, and bile leak in 11.5% of the patients. As for the ERCP types, biliary stent was placed in 46.2% of the patients, and nasobiliary drainage was performed in 38.5% of the patients. The demographic and clinical characteristics of the patients are shown in **Table 1**.

**Table 1:** Baseline characteristics of enrolled patients.

Variables		
**Age, mean, SD, y**	48.7	11.1
**Gender**		
Female, n, %	3	11.5%
Male, n, %	23	88.5%
**BMI, mean, SD, kg/m^2^**	21.5	3.1
**Time after liver transplantation, median, IQR, month**	10.0	2.8–44.3
**Indication for procedure**		
Anastomotic stricture, n, %	23	88.5%
Bile leak, n, %	3	11.5%
**ERCP types**		
Biliary stent placement, n, %	12	46.2%
Nasobiliary drainage, n, %	10	38.5%
Others, n, %	4	15.4%
**Liver function**		
ALT, mean, SD, U/L	54.0	48.0
AST, mean, SD, U/L	66.2	93.8
ALP, mean, SD, U/L	323.5	392.2
GGT, mean, SD, U/L	385.9	280.8
TBA, mean, SD, μmol/L	82.6	120.5
TBIL, mean, SD, μmol/L	107.7	124.3
DBIL, mean, SD, μmol/L	86.0	97.4
**Immunosuppressive regime**		
Tacrolimus only, n, %	12	46.2
Tacrolimus + MMF/corticoid, n, %	14	53.8
**Daily tacrolimus dose, mean, SD, mg**	3.1	1.9
**TAC_min_ pre-ERCP, mean, SD, ng/ml**	5.8	2.5

BMI, body mass index; ALT, alanine transaminase; AST, aspartate aminotransferase; ALP, alkaline phosphatase; TBI, total bilirubin; MMF, mycophenolate sodium enteric-coated tablets or mycophenolate mofetil.

### Variation in TAC C_min_

**Figure 2:**
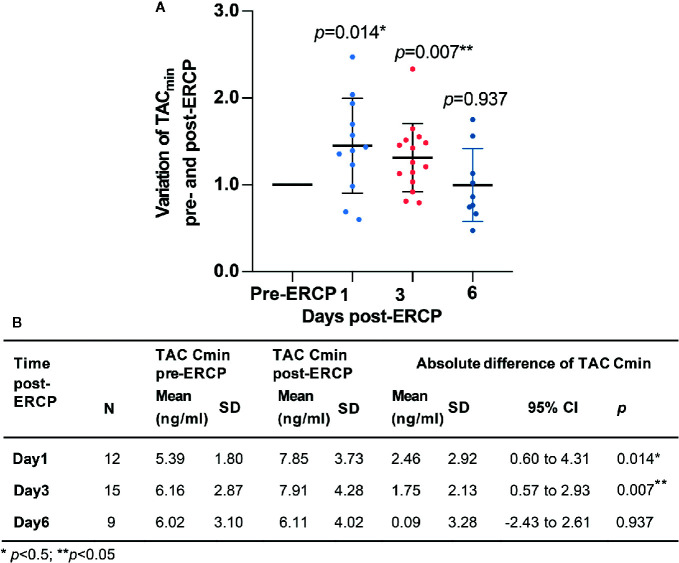
Variation of TAC C_min_ pre- and post-ERCP. **(A)** Extent of variation in TAC C_min_ pre- and post-ERCP (Standard TAC C_min_). Bars represent mean ± SD. **(B)** Absolute variation of TAC C_min_ pre- and post-ERCP.

A total of 73 eligible TAC C_min_ values were collected from the clinical electronic medical records. Among them, 26 TAC C_min_ values were tested pre-ERCP, 12, 4, 15, 3, 4, and 9 TAC C_min_ values were tested on days 1–6 post-ERCP, respectively. The mean C_min_ of TAC was 7.85 (SD: 3.73) and 7.91 ng/ml (SD: 4.28) on the first and third days post-ERCP, respectively. Both values were significantly higher than the TAC C_min_ pre-ERCP (*p*<0.05, **Figure 2**). The mean extent of variation in TAC C_min_ was 45.1% (95% CI: 28.3–81.3%, **Figure 2**) and 31.4% (95% CI: 9.7–53.1%, **Figure 2**) on days 1 and 3 post-ERCP, respectively. The increasing TAC C_min_ gradually returned to baseline on the sixth day post-ERCP (*p*>0.05, **Figure 2**). The inter-individual difference of the variation extent in TAC C_min_ was high. On the first and third day post-ERCP, the extent of variation in TAC C_min_ was over 50% in 41.7% and 26.7% of the patients, respectively, and even over 100% in two patients on the first day post-ERCP.

### Characteristics of Patients in Different Groups

The TAC C_min_ increased by more than 20% in 18 out of 26 (69.2%) patients during the first three days post-ERCP. Patients in the obvious variation group received significantly higher TAC dose pre-ERCP (*p*=0.001, **Table 2** and **Supplement Table 1**). The total bile acid (TBA) level was 113.7 (SD: 134.1) μmol/L in patients with obvious variation in TAC C_min_, compared with 12.6 (SD: 9.8) μmol/L in patients with mild variation (*p*=0.005). Although we observed a trend towards increased direct bilirubin (DBIL) and γ-glutamyltranspeptidase (GGT) levels in patients with obvious variation in TAC C_min_, the difference did not reach statistical significance (*p*>0.05, **Table 2**). The difference in other demographics, clinical characteristics, variation in laboratory data, indications for ERCP, and ERCP types between the two groups was not significant (*p*>0.05, **Tables 2** and **3**). Serum amylase levels were similar between the two groups (*p=*0.455) on the first day post-ERCP, and none of the patients developed pancreatitis.

**Table 2:** Comparation of demographics and clinical characteristics between patients with or without obvious variation of TAC C_min_.

Variables	Mild variation group (n=8)	Obvious variation group(n=18)	*p*
**Age, mean (SD), y**	50.0 (13.1)	48.2 (10.5)	0.707
			0.667
<45, n (%)	3 (37.5%)	5 (38.5%)	
≥45, n (%)	5 (62.5%)	13 (72.2%)	
**Sex**			0.529
Male, n (%)	8 (100%)	15 (83.3%)	
Female, n (%)	0 (0%)	3 (16.7%)	
**BMI**	22.7 (2.1)	21.0 (3.3)	0.221
**Times after LT, median, [IQR], month**	21 (1.0–79.5)	7 (3.8–42.5)	0.673
**Indication for procedure**			0.529
Anastomotic stricture, n (%)	8 (100%)	15 (83.3%)	
Bile leak and others, n (%)	0 (0%)	3 (16.7%)	
**ERCP Type**			0.730
Biliary stent placement, n (%)	3 (37.5%)	9 (50.0%)	
Nasobiliary drainage, n (%)	3 (37.5%)	7 (38.9%)	
Others, n (%)	2 (25.0%)	2 (11.1%)	
**Immunosuppressive regime**			0.401
Tacrolimus only, n (%)	5 (62.5%)	7 (38.9%)	
Tacrolimus + MMF/corticoid, n (%)	3 (37.5%)	11 (61.1%)	
**Dose of TAC, mean (SD), mg**	1.7 (0.8)	3.8 (1.9)	0.001^**^
**TAC C_min_ pre-ERCP, mean (SD), ng/ml**	4.5 (1.8)	6.4 (2.6)	0.060
**Laboratory data pre-ERCP**			
AST, mean (SD), U/L	43.1 (37.1)	76.4 (109.5)	0.414
ALT, mean (SD), U/L	44.6 (33.8)	58.2 (53.5)	0.518
TBIL, mean (SD), μmol/L	56.0 (71.8)	130.6 (137.1)	0.162
DBIL, mean (SD), μmol/L	42.0 (57.5)	105.5 (106.2)	0.061
ALP, mean (SD), U/L	188.8 (146.3)	383.4 (452.8)	0.251
GGT, mean (SD), U/L	228.6 (180.1)	455.7 (292.9)	0.055
TBA, mean (SD), μmol/L	12.6 (9.8)	113.7 (134.1)	0.005^**^
Scr, mean (SD), μmol/L	72.1 (19.2)	67.7 (27.7)	0.689
CRP, mean (SD), mg/L	25.7 (29.5)	24.3 (33.0)	0.925
Uric acid, mean (SD), μmol/L	250.8 (122.9)	242.8 (132.3)	0.887
**Serum amylase**			
6h after ERCP, mean (SD), U/L	220.8 (348.8)	250.3 (347.1)	0.843
12h after ERCP, mean (SD), U/L	145.0 (186.9)	250.6 (368.8)	0.455

ALT, alanine transaminase; AST, aspartate aminotransferase; ALP, alkaline phosphatase; TBI, total bilirubin; Scr, serum creatinine; CRP, serum c-reactive protein; MMF, mycophenolate sodium enteric-coated tablets or mycophenolate mofetil; **p < 0.01.

**Table 3:** Variation of laboratory data in patients with or without obvious variation of TAC C_min_.

Variation of laboratory data	Mild variation group (n=8)	Obvious variation group(n=18)	*p*
AST, mean (SD), U/L	4.5 (16.2)	−6.3(141.9)	0.833
ALT, mean (SD), U/L	−5.6 (19.3)	−3.6 (82.2)	0.945
TBIL, mean (SD), μmol/L	31.7 (96.8)	−10.5 (181.5)	0.545
DBIL, mean (SD), μmol/L	23.8 (79.5)	1.2 (135.5)	0.667
ALP, mean (SD), U/L	0.6 (57.2)	39.4 (861.9)	0.901
GGT, mean (SD), U/L	−22.6 (89.7)	−22.0 (378.9)	0.996
TBA, mean (SD), U/L	36.3 (110.3)	−59.2 (163.9)	0.147
Scr, mean (SD), μmol/L	17.0 (20.2)	16.8 (27.4)	0.984
CRP, mean (SD), mg/L	−0.8 (21.7)	3.2 (32.2)	0.768
Uric acid, mean (SD), μmol/L	66.3 (68.0)	49.4 (62.2)	0.542
Plasma potassium, mean (SD), mmol/L	0.6 (0.7)	0.3 (0.5)	0.316
Plasma sodium, mean (SD), mmol/L	−2.4 (2.9)	−2.8(3.6)	0.828

ALT, alanine transaminase; AST, aspartate aminotransferase; ALP, alkaline phosphatase; TBI, total bilirubin; Scr, serum creatinine; CRP, serum c-reactive protein.

## Discussion

Factors interfering with TAC exposure *in vivo* are complicated. Information about these factors is limited and insufficient, possibly leading to high interindividual and intraindividual variabilities in TAC exposure. For the first time, we described the variation regularity of TAC C_min_ during a common interventional procedure in LT patients, after excluding possible interferences. The TAC C_min_ value showed a significant increase (35.8–45.1%) during the first three days post-ERCP, and the increasing TAC C_min_ gradually returned to baseline on the sixth day post-ERCP. Considering that LT patients with biliary complications may require several ERCPs, high variability in TAC exposure during such procedures may increase TAC-related toxicity and risks of graft injury caused by the accumulation of subclinical rejections (Shemesh et al., 2017; Del Bello et al., 2018; Defrancq et al., 2019; van der Veer et al., 2019; Kuypers, 2020). Therefore, researchers should pay attention to this phenomenon. TAC dose pre-ERCP and TBA levels may be related to the variation extent in TAC C_min_. The findings may help elucidate the complex factors interfering with TAC exposure and optimize the TAC dose in clinical practice.

After searching the Medline database systematically, we found that reports about the influence on TAC exposure from surgeries-related or interventional procedures-related factors are rare. Possible interfering factors (Vanhove et al., 2016; Brunet et al., 2019), such as diarrhea pre-ERCP, dose adjustments, and newly added concomitant medicines (several kinds of proton pump inhibitors, triazole antifungal medicine, and calcium channel blockers) were excluded at first. The TAC C_min_ value significantly increased during the early phase post-ERCP, and returned to baseline level subsequently. The process coincided with the time of ERCP and indicated that procedures or procedure-related factors were associated with the exposure variation in TAC.

TAC dose pre-ERCP, rather than demographic characteristics, indication for ERCP, and ERCP types, was related to the variation extent in TAC C_min_ during the ERCP process. To prevent post-ERCP pancreatitis, we asked the patients to fast for approximately 24 h after ERCP in our center, which is a practice commonly conducted in other centers (Barthet et al., 2002; Ferreira et al., 2010; Park et al., 2018). The long fasting time may improve TAC absorption. The effect of mealtime on TAC bioavailability was studied previously. Fasting for 10 h provided higher relative bioavailability of TAC than ingestion of TAC 1 hour before or 1.5 h after the meal (Ferreira et al., 2010). High TAC dose may provide additionally growing potential for TAC C_min_ during a relatively long fasting period after ERCP.

Another factor related to the variation in TAC C_min_ was the TBA level pre-ERCP. It has been reported that in a mass balance study of intravenously administered radiolabeled TAC to 6 healthy volunteers, the fecal elimination accounted for 92.4 ± 1.0% of radioactivity (Astellas Pharma US Inc, 2015), suggesting liver clearance is the major elimination pathway of TAC. Consistently, bile acids underwent enterohepatic recirculation *via* hepatic transporters and greatly associated with liver function (Dawson et al., 2009). The effects on bile acid metabolism from TAC were investigated in previous studies (McCashland et al., 1994; Ericzon et al., 1997), but the relationship between TBA and TAC disposition was not reported. However, a series of reports found that the clearance of TAC was negatively correlated with bilirubin levels (Jacobson et al., 2001; Staatz and Tett, 2002; Lee et al., 2006; Campagne et al., 2019). For example, in a cohort study of 122 bone marrow transplant patients (Jacobson et al., 2001), the TAC clearance was approximately 40% lower in patients with bilirubin levels exceeding 171 µmol/l. The underlying mechanism is still unclear, and researchers hypothesized that the impaired liver function or biliary tract dysfunction affected the clearance of TAC (Jacobson et al., 2001; Lee et al., 2006; Vanhove et al., 2016). Our results showed high trends of DBIL and GGT levels in patients in the obvious variation group. Therefore, we speculated that the reduced clearance of TAC due to biliary tract dysfunction improves the TAC C_min_ post-ERCP. Although the variation in TAC C_min_ is not related to several adverse event indicators, such as serum amylase levels, variation in electrolyte level, or the variation in liver function indicators, the TAC concentration should be monitored frequently in patients with high TAC dose or high TBA levels to avoid potential risks of graft injury caused by the accumulation of subclinical rejections.

Our study had some limitations. First, this study was a retrospective, case series study with small sample size. After excluding data with potential interference by other known factors, the eligible data became even more limited, so that we are unable to further investigate whether such a variation of TAC C_min_ would increase the risk of sub-acute rejection. However, these data reflected real-world clinical practices and provided relevant information about the variation regularity of TAC C_min_. Second, as a retrospective study, the area under the concentration–time curve, which is considered the best pharmacokinetics parameter associated with the clinical effects of TAC (Brunet et al., 2019), cannot be achieved from the electronic medical records. TAC C_min_ is a good indicator of the exposure of TAC, and C_min_-guided therapy is recommended in many important guidelines (EASL, 2016; Charlton et al., 2018). Therefore, the variation regularity of TAC C_min_ can represent the exposure degree of TAC to a certain extent. Lastly, our results may not be extrapolated to other interventional procedures or surgery procedures directly considering that different surgery procedures possess various characteristics. Future research should focus on other interventional procedure-related factors and explore the specific mechanism involved in increased TAC C_min_ post-ERCP.

## Conclusion

TAC C_min_ significantly increased during the first three days post-ERCP in LT patients and the increasing TAC C_min_ gradually returned to the basic levels within a week. Such a variation may be correlated with TAC dose and TBA levels pre-ERCP but may not result in serious adverse events. The TAC C_min_ should be closely monitored during ERCP, especially in LT patients with high TAC dose and biliary tract dysfunction.

## Data Availability Statement

Data sharing on reasonable request can be available after approval from the corresponding authors. Only data without any identifiers will be made available.

## Ethics Statement

The studies involving human participants were reviewed and approved by the Ethics Committee of the First Affiliated Hospital, College of Medicine, Zhejiang University. Written informed consent for participation was not required for this study in accordance with the national legislation and the institutional requirements.

## Author Contributions

TL and WZ participated in the conception and design of this study. RW and WW were the project managers and coordinated patient recruitment. KM, XD, and RW coordinated all analysis of the project. RW, WW, FW, and MH were involved in the acquisition, analysis, or interpretation of data. Data analysis was done by MH and XD. Drafting of the manuscript was done by RW, WZ, and KM. All authors contributed to the article and approved the submitted version.

## Funding

This work was supported by the National Natural Science Foundation of China [81803502] and the Natural Science Foundation of Zhejiang Province [LYY20H310001].

## Conflict of Interest

The authors declare that the research was conducted in the absence of any commercial or financial relationships that could be construed as a potential conflict of interest.

## Supplementary Material

Supplement Table 1 Extent of variation in TAC C_min_ of included patients within 3 days post-ERCP. ^*^The TAC C_min_ and relative dose of TAC post-ERCP. If there were more than one value, the max value of TAC C_min_ was shown.
