# Supplementary material for: Variation in Tacrolimus Trough Concentrations in Liver Transplant Patients Undergoing Endoscopic Retrograde Cholangiopancreatography: A Retrospective, Observational Study
Source: Front Pharmacol. 2020 Aug 19;11:1252. doi: 10.3389/fphar.2020.01252 (PMC7466563; doi:10.3389/fphar.2020.01252)
Supplement: Supplement Table 1 — Extent of variation in TAC Cmin of included patients within 3 days post-ERCP. *The TAC Cmin and relative dose of TAC post-ERCP. If there were more than one value, the max value of TAC Cmin was shown. [file Table_1.docx]

Supplement Table 1 Extent of variation in TAC C_min_ of included patients within 3 days post-ERCP.

| No | Dose(mg/day) | Extent of variation in TAC C_min_ >20% within 3 days post-ERCP | C^*^/Dose/(C_pre-ERCP/_ Dose_pre-ERCP_) |
| --- | --- | --- | --- |
| 1 | 0.5 | No | 0.83 |
| 2 | 1.0 | Yes | 1.57 |
| 3 | 1.0 | No | 0.98 |
| 4 | 1.0 | No | 0.69 |
| 5 | 1.5 | Yes | 1.70 |
| 6 | 1.8 | No | 0.81 |
| 7 | 2.0 | No | 0.92 |
| 8 | 2.0 | Yes | 2.33 |
| 9 | 2.0 | No | 1.14 |
| 10 | 2.0 | Yes | 1.42 |
| 11 | 2.0 | Yes | 1.23 |
| 12 | 2.0 | Yes | 1.94 |
| 13 | 2.5 | Yes | 1.21 |
| 14 | 2.5 | No | 1.13 |
| 15 | 3.0 | Yes | 2.04 |
| 16 | 3.0 | No | 0.60 |
| 17 | 4.0 | Yes | 1.36 |
| 18 | 4.0 | Yes | 1.46 |
| 19 | 4.0 | Yes | 1.44 |
| 20 | 4.0 | Yes | 1.52 |
| 21 | 4.0 | Yes | 1.65 |
| 22 | 5.5 | Yes | 2.47 |
| 23 | 6.0 | Yes | 1.39 |
| 24 | 6.0 | Yes | 1.75 |
| 25 | 6.0 | Yes | 1.48 |
| 26 | 8.0 | Yes | 1.26 |

^*^The TAC C_min_ and relative dose of TAC post-ERCP. If there were more than one value, the max value of TAC C_min_ was shown.
